# Analysis of the Reasons for Medical Malpractice Litigation Due to Facet Injections

**DOI:** 10.7759/cureus.35015

**Published:** 2023-02-15

**Authors:** Haad Arif, Jacob Razzouk, Daniel Bohen, Omar Ramos, Olumide Danisa, Wayne Cheng

**Affiliations:** 1 Medicine, University of California Riverside School of Medicine, Riverside, USA; 2 Medicine, Loma Linda University School of Medicine, Loma Linda, USA; 3 Biomedical Engineering, University of Southern California Viterbi School of Engineering, Los Angeles, USA; 4 Orthopaedic Surgery, Twin Cities Spine Center, Minneapolis, USA; 5 Orthopedic Surgery, Loma Linda University Medical Center, Loma Linda, USA; 6 Orthopedic Surgery, Jerry L Pettis Memorial Veterans Hospital, Loma Linda, USA

**Keywords:** verdictsearch, westlaw legal database, litigation, malpractice, facet joint injection

## Abstract

Introduction

As the use of facet joint injections (FJI) increases, practitioners performing FJI may be at increased risk of legal liability. Malpractice claim analysis is performed by several specialties as it provides valuable insight into patient values and methods to mitigate the risk of malpractice litigation pertaining to a specific procedure or treatment. Malpractice analysis regarding FJI may provide clinicians with a better understanding of the reasons that lead to malpractice due to FJI, thereby allowing practitioners to improve the quality of care delivered to patients whilst mitigating the incidence of malpractice. The aim of our study was to analyze the reasons for malpractice litigation due to FJI by querying Westlaw and VerdictSearch, two well-established legal databases widely used in medicolegal research.

Methods

We queried the Westlaw Edge and VerdictSearch legal databases utilizing the terms "facet injection" and "spine". Our database queries yielded 1026 results on Westlaw Edge and 545 results on VerdictSearch. Cases were reviewed and categorized by two independent reviewers based on the grievance(s) levied by the plaintiff. Discrepancies between reviewers were resolved by a third reviewer. Inclusion criteria for case relevance were defined as a basis of litigation resting on malpractice claims filed between the years 2000-2022 directly pertaining to FJI. Additional data collected included the date of the case hearing, verdict ruling, location of filed claim, payment or settlement amount, and sustained injuries.

Results

Of all 1571 cases reviewed, 1568 cases were excluded due to a basis of litigation unrelated to FJI. Of the three cases pertaining to FJI, the first case involved an alleged procedural error on the part of the anesthesiologist, whereby the anesthesiologist misplaced the needle during FJI, resulting in quadriplegia due to a cervical spine infarction. The plaintiff also contended that the pre-procedural timeout was improperly conducted as the practitioner utilized iohexol as the injected contrast material despite the patient's well-documented allergy to iohexol. The jury deemed both the practitioner and hospital negligent, and a plaintiff verdict was issued. The second case was filed under a basis of litigation alleging delayed diagnosis and treatment on the part of an emergency medicine physician. The court acquitted the physician, and a defense verdict was issued. The third case was filed under a basis of litigation of alleged deviation from the standard of care on the part of the anesthesiologist, whereby the anesthesiologist performing the FJI did not use fluoroscopy. The court affirmed fluoroscopy is not dictated as the standard of care for FJI and issued a defendant verdict.

Conclusion

This study provides insight into the risk of medical malpractice suits brought on by facet joint injection. Our findings suggest that despite the high prevalence of facet joint injections performed annually, there is limited legal liability associated with the procedure. Nevertheless, there are certain reasons a malpractice claim may be filed due to facet injection, including gross procedural error resulting in patient paralysis, delay in treatment or diagnosis, and deviation from the established standard of care. As such, treatment decisions regarding facet joint injection should not be influenced by medicolegal concerns and remain predicated on patient care needs and standard of care.

## Introduction

Administration of facet joint injections (FJI) increased by over 300% from 2000 to 2014 [1]. While largely regarded as a safe procedure, FJI is not an entirely risk-free intervention. As the use of FJI increases, practitioners performing FJI may be at increased risk of legal liability. Malpractice claim analysis has been performed by several specialties as it provides valuable insight into patient values and methods to mitigate the risk of malpractice litigation pertaining to a specific procedure or treatment [2-9]. Malpractice claims analysis regarding FJI may provide clinicians with a better understanding of the reasons that lead to malpractice litigation, thereby allowing practitioners to improve the delivery of quality care to patients whilst mitigating the incidence of malpractice. The aim of our study was to analyze the reasons for malpractice litigation due to FJI by querying Westlaw (Thomson Reuters, Toronto, Canada) and VerdictSearch (ALM, New York City, New York), two well-established legal databases widely used in medicolegal research [2-9].

## Materials and methods

We queried the Westlaw Edge (Thomson Reuters, MN) and VerdictSearch (ALM Media Properties, NY) legal databases utilizing the terms "facet injection" and "spine". Westlaw Edge and VerdictSearch are considered to be two of the largest legal databases and have been widely used for the purpose of medicolegal research. Westlaw Edge is comprised of over 40,000 smaller legal databases, and VerdictSearch is a database encompassing over 250,000 cases evaluated in United States federal as well as state courts. Additionally, VerdictSearch includes cases from all categories of litigation aside from criminal law, while Westlaw Edge is fully comprehensive in its catalog and also includes international cases outside of the United States federal and state legal systems. Despite their large catalogs, however, both Westlaw Edge and VerdictSearch are not all-inclusive and are unable to capture cases settled outside of the judicial system or those resolved without formal registration [10]. Nevertheless, Westlaw Edge and VerdictSearch are still considered to be leading commercial providers of legal cases within the professional legal community and have been extensively validated for use in legal research across several medical specialties [11-13]. 

**Figure 1:**
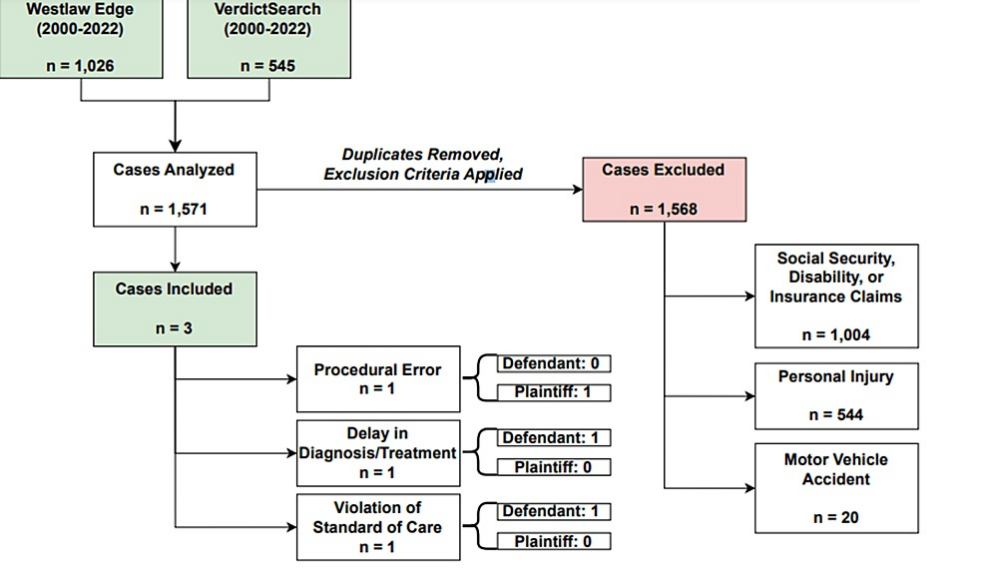
Review of malpractice claims from Westlaw Edge and VerdictSearch databases

Our database queries yielded 1026 results on Westlaw Edge and 545 results on VerdictSearch. As Westlaw and VerdictSearch contain overlapping case content, database results were screened to remove duplicates (Figure 1). Cases were reviewed and categorized by two independent reviewers based on the grievance(s) levied by the plaintiff. Discrepancies between reviewers were resolved by a third reviewer. While a broad keyword search was applied so as to capture the largest number of potential pertinent cases for review and possible inclusion in our study, any case mentioning the terms "facet joint injection" and "spine" were included in our review. Correspondingly, many cases in our review contained reasons for litigation unrelated to FJI and needed to be excluded from our study. Inclusion criteria for case relevance were defined as a basis of litigation resting on malpractice claims filed between the years 2000-2022 directly pertaining to FJI. Additional data collected included the date of the case hearing, verdict ruling, location of filed claim, payment or settlement amount, and sustained injuries. Data collection was performed using Microsoft Excel version 16.58 (Microsoft, Redmond, Washington). Descriptive statistics for case data comprised of percentage (%) values, mean values, and standard deviation values.

## Results

A total of 1571 cases were reviewed. Of all cases reviewed, 1568 cases were excluded due to a basis of litigation unrelated to FJI (Table 1). Of the three cases pertaining to FJI, the first case was filed in 2014 in California by a 64-year-old male plaintiff (Table 2). The basis of the litigation alleged procedural error on the part of the anesthesiologist, whereby the anesthesiologist misplaced the needle during FJI, resulting in quadriplegia due to a cervical spine (C2) infarction. The plaintiff also contended that the pre-procedural timeout was improperly conducted as the practitioner utilized iohexol as the injected contrast material despite the patient's well-documented allergy to iohexol. The jury deemed both the practitioner and hospital negligent: the practitioner and hospital were deemed 60% and 40% at fault, respectively. The plaintiff was awarded damages totaling $7,978,185.44: $176,201.29 in past economic damages, approximately $1,500,000 million for future hospitalizations, approximately $1,900,000 million for other future medical expenses, approximately $3,000,000 million for pain and suffering, and approximately $1,500,000 million for loss of consortium.

**Table 1:** Categorization of reviewed cases unrelated to FJI per basis of litigation FJI - facet joint injections

Basis of litigation (n=1568)	Category description
Social security, disability insurance, worker's compensation (n=1004)	Basis for litigation was to appeal for disability benefits or for damages due to injuries sustained while at work.
Personal injury (n=544)	Basis for litigation included injuries stemming from premise liability, negligent repair, or assault and battery.
Motor vehicle accident (n=20)	Basis for litigation was related to motor vehicle accident.

**Table 2:** Categorization of malpractice claims due to FJI per basis of litigation FJI - facet joint injections

Basis of litigation (n=3)	Category description
Injury due to an alleged procedural error during FJI (n=1)	Basis for litigation was an injury sustained following a needle misplacement during FJI.
Alleged delayed diagnosis/treatment of epidural hematoma following FJI (n=1)	Basis for litigation was delayed diagnosis and treatment of an epidural hematoma following FJI.
Alleged violation of the standard of care (n=1)	Basis of litigation was failure to use fluoroscopic guidance during FJI.

The second case was filed in April 2009 in Pennsylvania by a female plaintiff (the plaintiff's age was undisclosed). The basis of litigation alleged delayed diagnosis and treatment on the part of an emergency medicine physician. The plaintiff visited the emergency department due to numbness in her legs shortly after receiving FJI. The physician admitted the patient to the hospital for observation without ordering imaging. However, an MRI the following day revealed a large epidural hematoma compressing the spinal cord and necessitating emergency surgery. The plaintiff alleges that the defendant failed to properly evaluate and treat her symptoms despite her physical findings and recent history of FJI, causing a delay in diagnosis and contributing to lasting pain and inhibition of customary activities. The court deemed the medical provider nonnegligent, and a defense verdict was issued. 

The third case was filed in March 2017 in California by a 68-year-old female plaintiff. The basis of litigation rested on alleged deviation from the standard of care on the part of the anesthesiologist, whereby the anesthesiologist performing the FJI did not use fluoroscopy. The plaintiff opined that the lack of fluoroscopy resulted in a left posterior cervical cord lesion at C3-C4 and consequential neuropathic pain and disability. The defendant argued that 1) it was impossible for the 1.5-inch needle to reach the plaintiff's spinal cord, given the plaintiff's cervical spine anatomy, and 2) the use of fluoroscopy is not standard of care. The court affirmed fluoroscopy is not dictated as thee standard of care and ruled the anesthesiologist nonnegligent.

## Discussion

Malpractice claims analysis is performed by many specialties to provide insight into patients' values, methods to improve the quality of care, and risk factors for litigation pertaining to specific procedures or treatments. This study examined the incidence, reasons, characteristics, and outcomes of malpractice litigation due to FJI. Analyzing malpractice claims pertaining to FJI helps to characterize the general legal exposure of practitioners performing FJI as well as the specific reasons that may constitute malpractice due to FJI. A better understanding of the causes of medical malpractice litigation may help practitioners provide a higher quality of care to patients by minimizing the risk of committing malpractice. Our results demonstrate there are documented reasons for litigation following FJI, including gross procedural errors, delay in treatment or diagnosis, and deviations from standards of care. However, considering our study reviewed two of the largest commercial legal databases and found almost zero cases pertaining to FJI-specifically no class action lawsuits-this core finding of our study suggests liability related to FJI is minimal. As such, this study emphasizes that the practice of FJI, like all medical procedures, should not be influenced by medicolegal concerns and remain adherent to evidence-based standards of care.

While FJI is commonly associated with minor complications such as intravascular compromise and local bleeding, major complications of FJI are extremely rare but typically concern needle placement and drug administration that may result in nerve damage, spinal cord trauma, pneumothorax, or hematoma formation [14]. In 2012, Manchikanti et al. reviewed over 43,000 FJI and concluded that local hematoma was seen only in 1.2% of cases and that nerve root irritation was seen in only 0.02% of encounters [14]. Thus, while minimal, the risks of FI warrant caution to prevent patient injury and to decrease the incidence of malpractice. Our findings concur that although malpractice suits due to FJI are rare, there is documented risk of a malpractice claim being filed as a result of major neurological trauma. Previous studies have evaluated orthopedic, neurosurgical, and anesthetic pain management malpractice claims data, though none have focused specifically on FJI [15-18]. The present study revealed that two of the three FJI-related lawsuits resulted in a defense (physician-ruled) verdict. Nationally, 75% of spine-related lawsuits result in a defendant verdict, though there is considerable variation within the broad category [5]. For instance, in medical malpractice lawsuits involving incidental durotomies, 56.3% of court decisions result in a defendant ruling [6]. However, given our study's small sample size, this figure is but a mere observation and could very well be misleadingly inflated or underinflated. Our data overwhelmingly suggests that malpractice due to FJI is limited, but the existing data on the topic nevertheless demonstrates that the medical specialty of the provider consisted of anesthesiology in all FJI-related cases where the specialty was denoted. This may be due to the nature of the procedure itself as a form of pain management rather than purely a surgical intervention. Our study also highlighted that alleged procedural error during FJI is a claim that can result in a plaintiff ruling. Specifically, the alleged error consisted of the slippage of the needle during injection into the facet joint. Our study also revealed evidence that cases in which the plaintiff suffered from either permanent or temporary paralysis may also result in a plaintiff decision, which concurs with the findings of DePasse et al. [19]. Prior studies have found that an allegation of delayed diagnosis and treatment is significantly associated with a plaintiff court ruling [6]. This was not the case in our study, as both alleged delayed diagnosis, treatment, or deviations from the standard of care did not result in a plaintiff verdict. Furthermore, cases involving minor neurological injuries such as neuropathic pain, numbness, and weakness were not observed to result in a plaintiff verdict ruling. 

Limitations

Our study is not without several limitations. Though Westlaw Edge and VerdictSearch are the leading commercial providers of legal research both within the professional legal as well as medical communities, neither database captures all filed malpractice claims within the legal system. An estimated 72% of malpractice claims are dropped, denied, or dismissed prior to trial or settlement [20]. Correspondingly, there is potential for some malpractice claims due to FJI not being accessible for analysis as they are not part of formal judicial registration. As such, we by no means intend to make claims that our study is entirely comprehensive of all malpractice claims due to FJI. Rather, the cases included in our study likely represent only a sample of all malpractice claims due to FJI. A further limitation is not all court documents contained detailed patient medical histories, as medical jargon and granularity of detail vary on an unstandardized, case-by-case basis. This study is also limited due to its subjective nature, whereby the categorization of the cases reviewed was performed in a qualitative manner, with reviewers classifying cases based on the perceived interpretation of case details. While this subjectivity was, in part, accounted for by the use of multiple independent reviewers, there may be some degree of inherent variation in classification that stems from the study methodology. A final limitation of this study was its inability to draw significant statistical insight from the data due to the limited number of malpractice claims due to FJI. As such, the major finding of this study should be viewed as its inability to find cases due to FJI, suggesting limited legal liability associated with FJI. This is also in light of the fact that Westlaw and VerdictSearch have been validated to find several hundred cases pertaining to other procedures, and this study conducted a comprehensive review of over one thousand cases, using a broad keyword search to ensure a wide net was cast to identify potential cases [3, 7, 9]. All other findings should be viewed as merely descriptive and not indicative of any trends associated with FJI-related malpractice claims. 

## Conclusions

The findings of this study provide insight into the incidence of and reasons for medical malpractice claims filed due to facet joint injection. Our study reviewed two large, well-established legal databases and found few cases directly filed due to facet injection. Correspondingly, our study suggests that despite the high prevalence of facet joint injections performed annually, there is limited legal liability associated with the procedure. Nevertheless, there are certain reasons a malpractice claim may be filed due to facet injection, including documented instances of gross procedural error resulting in patient paralysis, alleged delay in treatment or diagnosis, and alleged deviation from the established standard of care. As such, treatment decisions regarding facet joint injection should not be influenced by medicolegal concerns and remain predicated on patient care needs and standard of care. 
